# Molecular Characterization of Human Respiratory Adenoviruses Infection in Xining City, China In 2018

**DOI:** 10.1007/s12250-020-00282-7

**Published:** 2020-09-14

**Authors:** Juan Yu, Shengcang Zhao, Huaxiang Rao

**Affiliations:** 1 Department of Basic Medical Sciences, Changzhi Medical College, Changzhi, 046000 China; 2 Department of Public Health and Preventive Medicine, Changzhi Medical College, Changzhi, 046000 China; 3 Center of Hygiene Inspection, Qinghai Center for Disease Control and Prevention, Xining, 810007 China; 4 Institute for Communicable Disease Control and Prevention, Qinghai Center for Disease Control and Prevention, Xining, 810007 China

Dear Editor,

Adenoviruses are non-enveloped, double-stranded DNA viruses belonging to the *Adenoviridae* family. To date, 100 unique genotypes of adenoviruses have been identified, from species A to G, could cause respiratory tract, gastrointestinal tract and ocular infections in human (Akello *et al.* 2020). Previous studies have reported that specific adenoviral types are often associated with certain clinical symptoms, epidemiological settings and demographic risk groups. Human adenovirus (HAdV) genotypes HAdV-B, HAdV-C, and HAdV-E are usually associated with respiratory infection; HAdV-A, -D, -F, and -G with gastrointestinal disease; and HAdV-D and E with ocular diseases in the healthy individuals (Lynch *et al.* 2011). Although HAdVs infection is mild and self-limited in the healthy individuals, it can be life-threatening in immune-compromised patients (Echavarria 2008).

HAdVs are playing an important role in the respiratory infections, which are responsible for several lower respiratory tract diseases. So far, little data on circulating HAdVs have been collected in Qinghai Province. Our previous study showed that the species HAdV-B3, HAdV-C1 and HAdV-C2 were the prevalent HAdV types in children in Xining City during 2016–2017 (Yu *et al.* 2019), which was a little different from the strains circulating in other provinces of China (Li *et al.* 1996; Xie *et al.* 2012; Zou *et al.* 2012).

The adenovirus capsid is composed of three major proteins: hexon, fiber and penton base. Based on the hypervariable regions, the *hexon* gene is most commonly used in the adenoviruses classification (Sarantis *et al.* 2004). Recently, phylogenetic analysis of the *fiber* gene has also been incorporated to observe the recombination events amongst adenovirus genotypes (Adhikary *et al.* 2011; Liu *et al.* 2011). In this study, we performed active surveillance for HAdV infections based on the *hexon* and *fiber* gene sequencing in Xining City. This knowledge would benefit for better understanding the prevalence and molecular evolution of HAdVs and might assist with the effective prevention and control of respiratory adenoviruses infection in Xining City.

As we know, from the influenza surveillance data, a large number of influenza-like cases were found negative for influenza virus. Adenoviruses can cause influenza-like illness, so we investigated the adenoviruses prevalence based on influenza surveillance network in our study. Nasopharyngeal swabs from patients with influenza-like illness (fever with body temperature more than 38 °C accompanied by respiratory symptom such as runny nose, sore throat, and cough) were collected from Qinghai Provincial People’s Hospital, Women’s and Children’s Hospital of Qinghai Province, and Qinghai Red Cross Hospital in 2018.

**Fig. 1:**
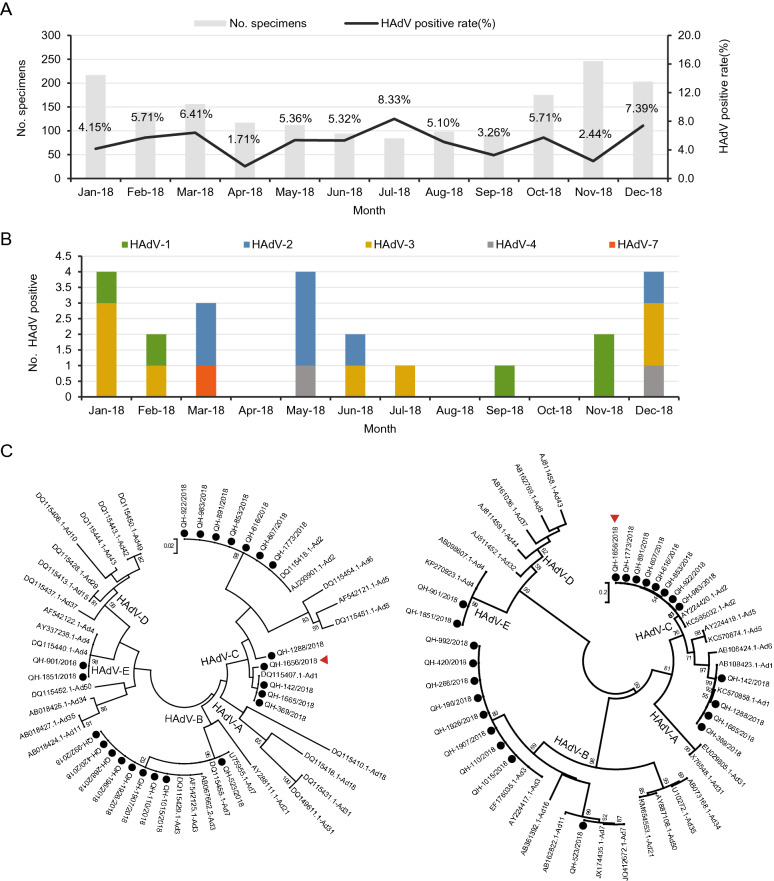
Prevalence of HAdVs in Xining City in 2018. **A** Monthly HAdV-positivity in nasopharyngeal specimens in 2018. **B** Monthly HAdV-strains isolated in 2018. **C** Phylogenetic tree based on partial *hexon* (left) and *fiber* (right) gene sequences of HAdV strains. Black dots represent Qinghai isolates, and red triangle represents the recombinant strain. Bootstrap proportions (1000 replications) are indicated as a percentage in each node.

In this study, a total of 1734 influenza-like cases with influenza virus-negative aged from 1 month to 95 years old were recruited, including 883 males and 851 females, 613 children and 1121 adults. Sampling intervals (from onset to nasopharyngeal swabs collection) were between 1 and 20 days. Then the samples were detected and isolated for HAdVs and performed *hexon* and *fiber* gene sequencing according to previous methods (Yu *et al.* 2019). The results showed that 86 of 1734 influenza-like cases were positive for HAdVs infection in 2018, the positive rate being 4.96%. Among that, positive rate of 4.64% (41/883) and 5.29% (45/851) were detected for males and females respectively, the difference was not statistically significant (*χ*^2^ = 0.382, *P *= 0.537). The median age of the positive cases was 9.5 years old, and the positive rate of 15.94% (11/69) was highest in the age of 4 years, followed by 12.00% in the age of 2 years (9/75), and the difference based on age was statistically significant (Fisher’s exact probabilities *P *< 0.001). In addition, the median interval time from the incidence of positive cases to sample collection were 2 (± 2) days, with the highest positive rate of 7.91% (17/215) at an interval of 3 days, followed by an interval of 4 days (6.98%), and the difference based on sampling intervals had no statistical significance (*χ*^2^ = 7.407, *P *= 0.192) (Table 1). During 2018, HAdVs infection was observed throughout the year, the positive rate of 8.33% in July was the highest and 1.71% in April was the lowest (Fig. 1A).

**Table 1:** Characteristics of gender, age and sampling intervals distribution of patients for HAdVs positive in Xining City in 2018.

Characteristic	Total samples (n)	HAdV positive samples (n)	Positive rate (%)	HAdV isolates (n)					*χ2/*Fisher exact probabilities	*P* value
				HAdV-1	HAdV-2	HAdV-3	HAdV-4	HAdV-7		
*Gender*										
Male	883	41	4.64	0	5	3	2	0	0.382	0.537
Female	851	45	5.29	5	2	5	0	1		
*Age(year)*										
< 0.5	47	4	8.51	1	0	0	0	0	–	< 0.001
0.5~	69	2	2.90	0	0	0	0	0		
1~	85	9	10.59	0	2	0	0	1		
2~	75	9	12.00	1	1	1	0	0		
3~	59	5	8.47	0	0	1	0	0		
4~	69	11	15.94	2	2	2	1	0		
5~	82	3	3.66	0	0	1	1	0		
10~	39	2	5.13	0	0	0	0	0		
15~	104	5	4.81	0	2	0	0	0		
20~	507	12	2.37	1	0	1	0	0		
40~	367	11	3.00	0	0	2	0	0		
60~95	231	13	5.63	0	0	0	0	0		
*Sampling intervals (day)*										
0~	230	9	3.91	0	1	2	0	0	7.407	0.192
1~	579	27	4.66	1	4	1	0	0		
2~	520	25	4.81	3	2	1	2	0		
3~	215	17	7.91	1	0	2	0	0		
4~	86	6	6.98	0	0	0	0	1		
5~20	104	2	1.92	0	0	2	0	0		
Total	1743	86	4.96	5	7	8	2	1

86 PCR positive samples were inoculated onto Hep-2 cell for virus isolation, and 23 HAdV strains were isolated through 7–21 days post-inoculation, with isolation rate of 26.74%, which was close to that of 21.43% in the previous study (Thounaojam *et al.* 2016). The *hexon* gene sequence has widely been used for the classification of adenovirus types. In this study, the *hexon* gene of the 23 HAdV isolates was amplified and sequenced using primer designed by Sarantis *et al.* (Sarantis *et al.* 2004) (Forward primer: 5′-CTGATGTACTACAACAGCACTGGCAACATGGG-3′, Reverse primer: 5′-GCGTTGCGGTGGTGGTTAAATGGGTTTACGTTGTCCAT-3′, the amplicon length is 580–605 bp), and the nucleotide sequences were submitted to GenBank (accession numbers MN389388–MN389410). Molecular typing assignments were based on the identity of the closest matching sequences after both BLAST and phylogenetic analysis. Results showed that 9 of the 23 HAdV isolates (8 HAdV-3 and 1 HAdV-7) belonged to species B and 12 of the 23 (5 HAdV-1 and 7 HAdV-2) belonged to species HAdV-C, and 2 of the 23 (2 HAdV-4) belonged to species HAdV-E. It revealed that HAdVs genotypes varied in different months and age groups (Fig. 1B, 1C; Table 1). Furthermore, the partial *fiber* gene was amplified and sequenced using primers designed by Xu *et al.* (Xu *et al.* 2000) (HAdV-B forward primer: 5′-TSTACCCYTATGAAGATGAAAGC-3′, reverse primer: 5′-GGATAAGCTGTAGTRCTKGGCAT-3′, the amplicon length is 670–772 bp; HAdV-C forward primer: 5′-TATTCAGCATCACCTCCTTTCC-3′, reverse primer: 5′-AAGCTATGTGGTGGTGGGGC-3′, the amplicon length is 1988–2000 bp; HAdV-E forward primer: 5′-TCCCTACGATGCAGACAACG-3′, reverse primer: 5′-AGTGCCATCTATGCTATCTCC-3′, the amplicon length is 967 bp), and the nucleotide sequences were submitted to GenBank (accession numbers MN389411–MN389433). The phylogenetic analysis of the *fiber* gene showed the same results for the HAdV-B and HAdV-E strains with *hexon* gene. However, phylogenetic analysis of the *fiber* gene showed that the results of four HAdV-1 and eight HAdV-2 in HAdV-C cluster were different from that of the *hexon* gene. It indicated that the recombination events might appear in the Qinghai adenovirus strains (Fig. 1C).

Acute respiratory tract illness is a major health problem globally, and about 5%–10% of acute respiratory infections are caused by adenoviruses (Thounaojam *et al.* 2016). Previous study showed that species B (HAdV-B3 and HAdV-B7), C (HAdV-C1, HAdV-C2, and HAdV-C5), and E (HAdV-E4) are usually associated with respiratory diseases (Lion 2014). Our study showed that the HAdVs prevalence was 4.96%, and prevalent throughout the year in 2018 in Xining City. Children at the age of 1–4 years were more susceptible to HAdVs infections, which was similar to the previous studies (Esposito *et al.* 2013; Cheng *et al.* 2017). HAdV-B3, -B7, HAdV-C1, -C2 and HAdV-E4 were prevalent in Xining City in 2018, among which HAdV-B7 and HAdV-E4 haven’t been reported before in this area (Yu *et al.* 2019). And, the most predominant isolates were HAdV-B3, HAdV-C1, and HAdV-C2, which were also the most commonly associated with respiratory HAdVs infection worldwide (Esposito *et al.* 2016). And the prevalence in 2018 was the same as prevalence during 2016–2017 in Xining City, which indicated that HAdVs infection might be relatively stable.

The identification of adenovirus genotypes is mainly determined by the sequence analysis of the *hexon* and *fiber* gene. New adenovirus genotypes are increasingly recognized, and some of the new types may acquire different pathogenicity and cause epidemic outbreaks (Lukashev *et al.* 2008). For example, HAdV-55, formed due to a recombination between HAdV-11 and HAdV-14 strains, is associated with acute respiratory disease outbreaks (Zhang *et al.* 2012). In this study, phylogenetic analysis showed one isolate clustered with HAdV-C1 based on *hexon* gene, but clustered with HAdV-C2 based on *fiber* gene, which indicated recombination events of HAdVs.

There are some limitations in this study. First, only 26.74% of all HAdV isolates were obtained and sequenced, which was lower than 56.25% in 2017 (Yu *et al.* 2019). Here, HAdVs strains were isolated through 7–21 days post-inoculation on Hep-2 cells for three times, but only 23 isolates were obtained, unfortunately no strains isolated in April, August and October. Second, the identification of recombinant strains was incomplete based on *hexon* and *fiber* genes, and the complete genome sequencing should be used for identifying the prevalence and the genetic variants of adenoviruses in the future. Third, the clinical characteristics of patients were missed because participants were all from outpatients in this study, as a result, the potential association between the genotypes of HAdVs, the clinical characteristics and the severity of patients could not be analyzed. In addition, HAdV-1, 2, 3, 4, 5, 6, 7 were observed in Xining City during 2 years of surveillance, but no HAdV-55 observed, which might be related to only outpatients being recruited in this study. This suggested that hospitalized patients should be included in further study.

In conclusion, HAdVs surveillance in Qinghai Plateau is limited. In 2018, HAdVs infection was observed in Xining City throughout the year, and HAdV-B3, HAdV-C1 and HAdV-C2 were predominant strains. In addition, one recombinant strain harboring the *hexon* gene of HAdV-C1 and *fiber* gene of HAdV-C2 was observed. Our study provided the molecular characteristics of HAdVs in Xining City, which indicated that HAdV recombinant should pay more attention and the corresponding prevention and control strategy should be taken into consideration in this area.
